# Effects of silicon on *Oryza sativa* L. seedling roots under simulated acid rain stress

**DOI:** 10.1371/journal.pone.0173378

**Published:** 2017-03-14

**Authors:** Shuming Ju, Ningning Yin, Liping Wang, Cuiying Zhang, Yukun Wang

**Affiliations:** 1 School of Environment and Spatial Informatics, China University of Mining & Technology, Xuzhou, Jiangsu, China; 2 Xuzhou Institute of Technology, Xuzhou, Jiangsu, China; Estacion Experimental del Zaidin, SPAIN

## Abstract

Silicon (Si) has an important function in reducing the damage of environmental stress on plants. Acid rain is a serious abiotic stress factor, and Si can alleviate the stress induced by acid rain on plants. Based on these assumptions, we investigated the effects of silicon on the growth, root phenotype, mineral element contents, hydrogen peroxide (H_2_O_2_) and antioxidative enzymes of rice (*Oryza sativa* L.) seedling roots under simulated acid rain (SAR) stress. The results showed that the combined or single effects of Si and/or SAR on rice roots depend on the concentration of Si and the pH of the SAR. The combined or single effects of a low or moderate concentration of Si (1.0 or 2.0 mM) and light SAR (pH 4.0) enhanced the growth of rice roots, and the combined effects were stronger than those of the single treatment. A high concentration of Si (4.0 mM) or severe SAR (pH 2.0) exerted deleterious effects. The incorporation of Si (1.0, 2.0 or 4.0 mM) into SAR with pH 3.0 or 2.0 promoted the rice root growth, decreased the H_2_O_2_ content, increased the Si concentration and the superoxide dismutase (SOD), catalase (CAT), peroxidase (POD) and ascorbate peroxidase (APX) activities, maintained the balance of mineral element (K, Ca, Mg, Fe, Zn, and Cu) concentrations in the roots of rice seedlings compared with SAR alone. The alleviatory effects observed with a moderate concentration of Si (2.0 mM) were better than the effects obtained with a low or high concentration of Si (1.0 or 4.0 mM). The observed effects were due to disruptions in the absorption and utilization of mineral nutrients and impacts on the activity of antioxidant enzymes in roots, and this conclusion suggests that the degree of rice root damage caused by acid rain might be attributed to not only acid rain but also the level of Si in the soil.

## Introduction

As a result of rapid worldwide economic growth, acid rain is becoming an increasingly serious environmental issue [1,2]. Along with Europe and North America, China has become a severely polluted region [3], and approximately 40% of its territory is affected by acid rain [4]. Although the average pH value of acid rain in China ranges from 3.0 to 4.5 [5], it can be as low as 1.3 [6]. Both the area affected by acid rain and the acidity of rainwater are increasing, and the frequency can reach a very high level [7]. The consequences of acid rain in plants include damage to the cell membrane system and negative impacts on respiration, photosynthesis, and the antioxidative enzyme system [8–11], and low yields, low germination rates, thin roots, premature abscission, branch dieback, necrosis, and morphological changes have been reported [3,8,12,13]. In addition, some studies have indicated that acid rain effects on the activities of superoxide dismutase (SOD), catalase (CAT) and peroxidase (POD) on plant species depend on the pH and duration of the acid rain treatments [9–11,14,15].

Silicon (Si), the second most abundant element in the terrestrial crust and soil, is beneficial for the healthy growth and development of many plant species [16–20]. In particular, the relationships of Si with resistance or tolerance to abiotic stress, including drought, high temperature, UV, nutrient loading, freezing, salinity, nutrient imbalance, and metal toxicity, have been studied extensively in many higher plants [17,21–27]. Si might be involved in metabolic, physiological and/or structural activities in higher plants exposed to abiotic and biotic stresses [28,29] and potentially mitigates the deleterious effects of acid rain, a serious abiotic stress.

Rice (*Oryza sativa* L.) is recognized as the second most consumed staple food for more than half of the world’s population [30,31]. As a model of a monocot plant, rice is also a typical Si-hyper-accumulating plant species, with the Si percentage in the roots reaching 10% on a dry-weight basis [7]. Previous results have shown that the application of Si is beneficial for rice. Si enhances bacterial blight resistance, reduces neck blast and lodging and increases yield in rice [32–36], which is known for its capability to actively absorb Si at high amounts [36]. The production of a total rice grain yield of 5000 kg·ha^-1^ will remove 230–470 kg·ha^-1^ Si from the soil; as a result, repeated mono-cropping with rice might greatly decrease the amount of Si in soil available to plants [37]. As a consequence, Si might subsequently become a yield-limiting element for rice cultivation [38–40], which is a staple grain crop in southern China, a region that is often affected by acid rain. Acid rain treatment could affect the growth of rice plants, and previous results have shown that rice is an acid-rain-tolerant plant [41–44]. The influence of acid rain on plants depends on the pH of SAR. Acid rain with low pH values can boost the rice yield [41]. SAR at pH 2–3.5 reduces the seed germination, water absorption, and respiration rates in rice [42,43]. However, Hosono and Nouchi showed that the rice yield is not affected by acid rain, even at a pH value of 2.5 [44]. However, to the best of our knowledge, few studies have investigated the combined effects of Si and acid rain on rice.

This study investigated the effects of Si on the root phenotype, growth, mineral element contents and activities of antioxidative enzymes of rice seedlings under SAR. The investigation aimed to understand the impacts and mechanisms of combined treatment with Si and SAR and to provide evidence demonstrating the biological and environmental roles of Si in enhancing tolerance to abiotic stress.

## Materials and methods

### Preparation of rice nutrient, SAR and Si solutions

A rice nutrient solution was prepared according to the ionic composition released by the International Rice Research Institute (IRRI) [45] with some modifications: 2.9 mM NH_4_NO_3_, 0.32 mM NaH_2_PO_4_, 1.0 mM K_2_SO_4_, 1.0 mM CaCl_2_, 1.7 mM MgSO_4_·7H_2_O, 36 μM FeCl_3_·7H_2_O, 18 μM H_3_BO_3_, 9.1 μM MnCl_2_·4H_2_O, 0.16 μM CuSO_4_·5H_2_O, 0.15 μM ZnSO_4_·7H_2_O, 0.52 μM (NH_4_)_6_MoO_4_·4H_2_O, and 77.42 μM citric acid. The pH of the nutrient solution was adjusted to 5.5 with 1 M NaOH or HCl using a PHS-29A pH metre (Shanghai Anting Scientific Instrument Factory, Shanghai, China). To obtain 1/4- or 1/2-strength nutrient solution, the macroelements were decreased to values equal to 1/4 or 1/2 of the initial values, but the Ca concentration remained unchanged.

SAR treatments with three pH values were used in this study. Specifically, SAR solutions with pH values of 4.0 (light acid rain), 3.0 (moderate acid rain) and 2.0 (severe acid rain) were prepared by adding ions to deionized water (Table 1), and a control rain solution at pH 6.5 was also prepared. The ionic composition was derived from precipitation data from eastern China [46,47]. The pH of the solution was adjusted to 6.5, 4.0, 3.0, and 2.0 using 1 mM H_2_SO_4_ and 1 mM HNO_3_ at a ratio of 2.7:1 based on chemical equivalents [48,49].

**Table 1:** Major ions of the SAR (mM).

pH	SO_4_^2-^	NO_3_^-^	NO_2_^-^	Cl^-^	F^-^	Ca^2+^	NH_4_^+^	Mg^2+^	K^+^	Na^+^
6.5	0.362	0.093	0.028	0.092	0.038	0.425	0.187	0.105	0.056	0.091
4.0	0.567	0.251	0.023	0.093	0.072	0.203	0.139	0.102	0.038	0.090
3.0	0.552	0.346	0.025	0.104	0.082	0.168	0.167	0.086	0.036	0.095
2.0	0.455	0.347	0.025	0.104	0.088	0.187	0.146	0.083	0.034	0.099

We also prepared four Si solutions with different Si concentrations: 1 mM (Low), 2 mM (Moderate), 4 mM (High) and 0 mM (control, CK). The Si solutions were prepared by dissolving the appropriate quantities of NaSiO·9H_2_O in IRRI nutrient solution.

### Plant culture and treatments

Rice seeds (Zhendao 95, Xuzhou Seed Co., Ltd., Xuzhou Jiangsu, China) were placed in a plastic net, covered with perlite (1.5-cm-thick layer), and germinated in an incubator at 25 ± 1°C. Once the height of the seedlings reached approximately 1 cm (day 6), the seedlings were transferred to 1/4-strength IRRI solution in the greenhouse at 25 ± 5°C. At 15 days of age, the seedlings were cultured in 1/2-strength IRRI solution, and at 24 days of age, the seedlings were cultured in full-strength IRRI solution and sprayed with the SAR solution. The Si treatments were initiated when the seedlings were six days of age. The solutions were replaced every three days, and water was added every day to ensure maintenance of the solution volume.

A full factorial experimental design with 16 treatment combinations was used. (1) The control treatment: rice seedlings were cultured in IRRI nutrient solution without Si (0 mM) and sprayed with SAR (pH 6.5) until drops began to fall from the foliage. (2) Single Si treatments: rice seedlings were cultured in IRRI nutrient solution with Si (1, 2 or 4 mM, pH 5.5) and then sprayed with SAR (pH 6.5) until drops began to fall from the foliage. (3) Single SAR treatments: rice seedlings were cultured in IRRI nutrient solution without Si (pH 5.5) and then sprayed with SAR (pH 4.0, 3.0 or 2.0) until drops began to fall from the foliage. (4) Combined Si and SAR treatments: rice seedlings were cultured in IRRI nutrient solution with Si (1, 2 or 4 mM, pH 5.5) and then sprayed with SAR (pH 4.0, 3.0 or 2.0) until drops began to fall from the foliage. Three replicates of each treatment were performed.

All of the plants were grown in a greenhouse with a natural photoperiod, and the relative humidity was maintained in the range of 50% to 70%. Rice seedlings treated with SAR for nine days were collected for the determination of various parameters, including parameters associated with the phenotype, growth, mineral element contents and antioxidative enzymes of the roots.

### Scanning and determination of root phenotype

A root automatism scanning apparatus (ScanMaker i800 plus) equipped with WinRHIZO software (LA-S series of plant image analysis system, Regent Instruments, Quebec, China) was used to determine the total root length (TRL), root tip number (RTN), root surface area (RSA), root volume (RV), and average diameter (AD). The aboveground parts of the rice seedlings were cut, and root segments were placed in a transparent plastic tray on the scanning apparatus. The lateral roots were stretched as far as possible without overlapping. Images were recorded at a resolution of 800 dpi. Various root phenotypic traits were evaluated using WinRHIZO LA-S software. Three roots from each of the seedlings from each treatment replicate were analysed.

### Determination of root fresh weight (FW) and dry weight (DW)

After nine days of treatment, the fresh roots of the rice seedlings were harvested, washed with distilled water and weighted. The fresh roots were then dried at 80°C for 12 h in an oven, and the DW of the roots was determined [50,51].

### Determination of mineral element contents

The mineral element and Si contents were determined according to Wang et al. [52] with some modifications. The fresh roots were collected, washed three times with distilled water, and dried in an oven at 80°C to a constant weight. Approximately 0.2 g of crushed roots from each sample was digested with a microwave digestion system (APL-MD6M, APL Instrument Co., Ltd., Chengdu, China) using 7 mL of oxidizing solution (6 mL of HNO_3_ and 1 mL of 30% H_2_O_2_, v/v) for 30 min (150°C for 10 min and 180°C for 20 min). The digested samples were diluted with deionized water to a final volume of 100 mL prior to analysis. The concentrations of Si and mineral elements (K, Ca, Mg, Fe, Zn, and Cu) in each digested solution were determined by inductively coupled plasma-optical emission spectroscopy (ICP-OES optima 8300, Perkin Elmer, MA, USA). The ICP-OES instrument was calibrated using standard solutions.

### Determination of SOD, CAT, POD and APX activities

Approximately 0.25 g of fresh roots was extracted in 50 mM potassium phosphate buffer (PBS, pH 7.8). The homogenates were centrifuged at 15,000×g and 4°C for 20 min, and the supernatants were used for assaying enzyme activity [53].

SOD (EC 1.15.1.1) activity was determined by measuring the inhibition of NBT photochemical reduction [54,55] with some modifications. Each 3-mL reaction mixture contained 2.2 mL of PBS (50 mM pH 7.8), 0.2 mL of Met (130 mM), 0.1 mL of EDTA-Na_2_ (20 μM), 0.2 mL of NBT (750 μM), 0.2 mL of riboflavin (100 μM), and 0.1 mL of the sample. The enzymatic activity is expressed as the amount of extract needed to inhibit the reduction of NBT by 50%.

CAT (EC 1.11.1.6) activity was measured by determining the decrease in the absorbance at 240 nm due to H_2_O_2_ consumption, as described by Azevedo et al. [56]. The reaction mixture contained 0.3 mL of H_2_O_2_ (0.1 M), 1.5 mL of PBS (50 mM pH 7.8), 0.2 mL of the sample and 1 mL of distilled water.

POD (EC 1.11.1.7) activity was determined according to Bai et al. [57] with some modifications. The reaction mixture contained 1.0 mL of 0.3% H_2_O_2_, 1.0 mL of 0.05 M PBS (pH 7.8), 0.9 mL of 0.2% guaiacol, and 0.1 mL of the sample. The increase in absorbance at 470 nm during a 1-min period was determined.

AXP (EC 1.11.1.11) activity was determined by measuring the decrease in the absorbance of ascorbic acid (ASA) at 290 nm [58,59]. The reaction mixture contained 2.7 mL of a mixture containing PBS (50 mM, pH 7.8) and AsA (1 mM), 0.1 mL of the sample, and 0.2 mL of H_2_O_2_ (4.5mM). The oxidation of ascorbate was initiated by H_2_O_2_ and was monitored for 1.5 min. One unit of APX activity was defined as the amount of enzyme oxidizing 1 μM of ascorbate, and the extinction coefficient was 2.8 mM^−1^cm^−1^.

### Determination of hydrogen peroxide (H_2_O_2_) content

H_2_O_2_ was determined according to Zhang et al. [11]. Approximately 0.5 g of fresh roots was homogenized in an ice bath with 3% (w/v) trichloroacetic acid (TCA). The homogenates were centrifuged at 12,000×g and 4°C for 15 min. Each 4-mL reaction mixture contained 1 mL of PBS (100 mM, pH 7.0), 1 mL of supernatant and 2 mL of KI (1 M). The absorbance at 390 nm was determined, and the content of H_2_O_2_ was calculated based on a standard curve.

### Statistical analysis

All data are presented as the means±SD. One-way analysis of variance (ANOVA) with least significant difference (LSD) was used to analyse the significance of the differences among different treatments using SPSS 19 and Origin 8.0. Two-way ANOVA was performed to test the interaction between Si and SAR. Student’s t-test was applied to compare the different treatments, and comparisons with *p*<0.05 were considered significantly different.

## Results

### Effects of Si on the root phenotype of rice seedlings under SAR

**Fig 1:**
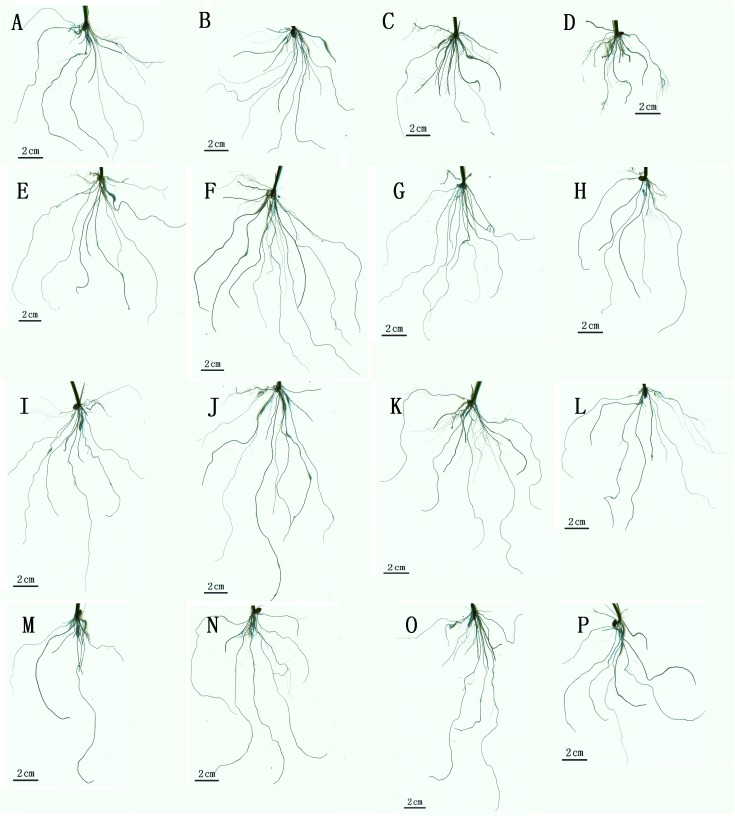
Images of rice seedling roots under different treatments. (A-D) no Si; (E-H) 1 mM Si; (I-L) 2 mM Si; (M-P) 4 mM Si; (A, E, I, M) no acid soil; (B, F, J, N) SAR at pH 4; (C, G, K, O) SAR at pH 3; and (D, H, L, P) SAR at pH 2.

Root phenotypic images of rice seedlings treated with Si and SAR are displayed in Fig 1, and data of the root phenotypic traits, including the total root length (TRL), root tip number (RTN), root surface area (RSA), root volume (RV), and average diameter (AD), are presented in Table 2. The results showed that the roots of the control rice seedlings were fully developed (Fig 1A).

**Table 2:** Effects of Si and SAR on root phenotypic traits of rice seedlings.

SAR(pH)	Si(mM)	TRL (cm)	RSA (cm^2^)	RV (cm^3^)	AD (cm)	RTN
6.5	0	60.605±2.328 de(100.00)	10.042±0.233 fgh(100.00)	0.265±0.013 hi(100.00)	0.621±0.010 fg(100.00)	181.40±7.27 ef(100.00)
	1	67.207±2.202 c(110.89)	12.307±0.723 de(122.56)	0.302±0.019 fg(113.96)	0.644±0.031 defg(103.70)	226.90±10.14 cd (125.08)
	2	66.596±2.557 c(109.89)	11.183±0.805 ef(111.36)	0.280±0.017 gh(105.66)	0.654±0.011 cde(105.31)	244.42±15.04 bc(134.74)
	4	46.280±1.688 g(76.36)	7.820±0.414 ij(77.87)	0.203±0.011 l(76.60)	0.745±0.023 a(119.97)	142.67±15.41 g (78.65)
4.0	0	70.375±2.198 c(116.12)	13.313±1.601 cd(132.57)	0.365±0.015 d(137.74)	0.647±0.009 gef(104.19)	217.07±12.39 d(119.66)
	1	93.057±1.096 a(153.55)	18.332±1.151 a(192.55)	0.507±0.028 a(191.32)	0.675±0.022 bc(108.70)	308.90±12.15 a (170.29)
	2	90.005±3.931 a(148.51)	17.424±0.242 a(173.51)	0.476±0.017 b(179.62)	0.683±0.011 b(109.98)	325.56±15.98 a(179.47)
	4	62.607±3.432 d(103.30)	10.155±0.800 fgh(101.13)	0.305±0.018 fg(115.09)	0.666±0.028 bcd(107.25)	189.86±18.46 e (104.66)
3.0	0	60.753±2.609 de(100.24)	11.457±0.678 def(114.09)	0.333±0.012 e(125.66)	0.636±0.007 efg(102.42)	176.20±17.12 ef(97.13)
	1	81.491±2.491 b(134.46)	15.123±1.054 b(150.60)	0.421±0.011 c(158.87)	0.654±0.019 cde(105.31)	235.67±11.23 cd(129.92)
	2	78.540±4.330 b(129.59)	14.078±1.001 bc(140.19)	0.396±0.014 c(149.43)	0.663±0.011 bcd(106.76)	262.06±17.54 b(144.47)
	4	58.261±2.308 ef(96.13)	10.259±0.625 fjh(102.16)	0.309±0.017 ef(116.60)	0.657±0.010 bcde(105.80)	170.76±13.52ef(94.13)
2.0	0	40.327±1.416 h(66.54)	6.485±0.663 j(64.58)	0.207±0.009 l(78.11)	0.720±0.013 a(115.94)	159.97±13.47 fg(88.19)
	1	57.525±2.629 ef(94.92)	9.454±0.826 gh(94.14)	0.233±0.013 jk(87.92)	0.633±0.005 fg(101.93)	184.57±16.96 e(101.75)
	2	60.281±1.583 de(99.47)	9.624±0.362 gh(95.84)	0.253±0.017 ij(95.47)	0.641±0.009 defg(103.22)	190.36±17.64 e(104.94)
	4	54.100±2.101 f(89.27)	9.130±0.687 hi(90.92)	0.211±0.003 kl(79.62)	0.617±0.006 g(99.36)	166.74±13.14 efg(91.92)
F		46.277	34.623	62.362	0.640	35.923
p		0.000^*^	0.000^*^	0.000^*^	0.698	0.000^*^

Abbreviations: SAR, simulated acid rain; Si, silicon; TRL, total root length; RSA, root surface area; RV, root volume; AD, average diameter; RTN, root tip number.

The values represent the means ± standard deviation, n = 3. The value in brackets is relative percentage (the ratio of absolute value to the control).

Significant differences (*p*<0.05) are indicated by different letters in the same column.

^*^ Significant at the 0.05 level.

The roots treated with 1 or 2 mM Si showed enhanced growth compared with the control (Fig 1E and 1I). Although these treatments significantly increased the TRL, RSA, RV and RTN in rice seedlings compared with the control treatment, the AD was unchanged (Table 2). In addition, the root phenotype and phenotypic traits of the seedlings subjected to the 1 mM and 2 mM Si treatments were note notably different. However, the roots of the rice seedlings treated with 4 mM Si were smaller than those of the control (Fig 1M); in addition, the TRL, RSA, RV and RTN observed in the 4 mM Si-treated seedlings were significantly decreased by 23.64%, 22.13%, 23.40% and 21.35%, respectively, whereas the AD was significantly increased by 19.97% (Table 2).

The root phenotype and phenotypic traits of the plants subjected to the SAR treatments were markedly different compared with those of the control plants. The roots subjected to the SAR (pH 4.0) treatment were larger than the control roots (Fig 1B), and the TRL, RSA, RV and RTN of the SAR (pH 4.0)-treated roots were significantly increased by 16.12%, 32.57%, 37.74% and 19.66%, respectively, but the AD was not changed (Table 2). The seedling roots treated with SAR at pH 3.0 also exhibited changes compared with the control (Fig 1C); in addition, compared with the control plants, the TRL, AD and RTN of the SAR pH 3.0-treated plants were not changed, but the RSA and RV were significantly increased by 14.09% and 25.66%, respectively (Table 2). In contrast, the SAR (pH 2.0)-treated roots were smaller compared than the control roots (Fig 1D). The TRL, RSA, RV and RTN of the roots subjected to this treatment were significantly reduced compared with the control by 33.47%, 35.42%, 21.89% and 11.81%, respectively, but the AD was increased by 15.94% (Table 2).

As shown in Table 2, the data obtained under SAR at pH 4.0, 3.0 or 2.0 revealed no obvious changes in the phenotype and phenotypic traits of roots subjected to the 1 mM Si treatment compared with the 2 mM Si-treated roots. However, the roots treated with 1 or 2 mM Si and SAR at pH 4.0 showed the best parameters, as indicated by the finding that the roots subjected to these combined treatments presented increases in the TRL, RSA, AD, RV and RTN compared with the roots treated with 1 or 2 mM Si alone and the roots subjected to the single treatment with SAR at pH 4.0. The roots treated with 1 or 2 mM Si and SAR at pH 3.0 exhibited significantly higher TRL, RSA and RV compared with the control, 1 or 2 mM Si-treated, and SAR (pH 3.0)-treated roots. However, the roots of rice seedlings treated with 1 or 2 mM Si and SAR at pH 2.0 were smaller and presented inferior phenotypic traits compared with those of seedlings subjected to the single 1 mM and 2 mM Si treatments; in contrast, these roots were larger and presented improved phenotypic traits compared with the roots of seedlings subjected to treatment with SAR at pH 2.0, with no obvious differences compared with the control. The combined treatment with the high concentration of Si (4 mM) and SAR at pH 4.0 had fewer deleterious effects on the root phenotype of rice seedlings in comparison with SAR at pH 4.0 alone, with no differences compared with the control, and the inhibitory effect associated with Si stress appeared to be decreased. The TRL, RTN, RSA and RV of the roots treated with 4 mM Si and SAR at pH 3.0 were significantly increased compared with the roots subjected to single treatment with 4 mM Si, but no significant changes were observed compared with the control and the single treatment with SAR at pH 3.0. Under severe SAR (pH 2.0), the high concentration of Si (4.0) appeared to relieve the effects of the SAR and 4 mM Si single stress treatments. In summary, in the presence of a high concentration of Si (4 mM), SAR had a lower impact on the roots of rice seedlings; similarly, Si relieved the effects of severe SAR (pH 2.0) stress. The results of a two-way ANOVA revealed an obvious interaction between Si and SAR that affected the TRL, RTN, RSA, AD and RV (Table 2).

### Effects of Si on the FW and DW of rice seedling roots under SAR

Table 3 shows the effects of Si on the FW and DW of rice seedling roots under SAR stress. The FW and DW of roots subjected to the 1 or 2 mM Si treatments were unchanged compared with the control (Table 3). However, the FW and DW of the roots treated with the high concentration of Si (4 mM) were decreased by 11.92% and 5.10%, respectively, compared with the control (Table 3).

**Table 3:** Effects of Si and SAR on the root FW and DW of rice seedlings.

SAR (pH)	Si (mM)	FW (g·stock^-1^)	DW (g·stock^-1^)
6.5	0	0.0856±0.0045 ef(100.00)	0.0098±0.0006 ef(100.00)
	1	0.0893±0.0030 e(104.32)	0.0101±0.0007 def(103.06)
	2	0.0889±0.0040 e(103.86)	0.0108±0.0006 cde(110.20)
	4	0.0754±0.0065 g(88.08)	0.0093±0.0010 fg(94.90)
4.0	0	0.0995±0.0049 d(116.24)	0.0116±0.0011 bc(118.37)
	1	0.1392±0.0036 a(162.62)	0.0157±0.0011 a(160.20)
	2	0.1233±0.0059 b(144.04)	0.0158±0.0009 a(161.22)
	4	0.0895±0.0041 e(104.56)	0.0114±0.0009 bcd(116.33)
3.0	0	0.0869±0.0050 e (101.52)	0.0102±0.0004 def(104.08)
	1	0.1151±0.0054 c(134.46)	0.0112±0.0014 bcd(114.29)
	2	0.1097±0.0069 c(128.15)	0.0123±0.0008 b(125.51)
	4	0.0889±0.0050 e(103.85)	0.0109±0.0004 cde(111.22)
2.0	0	0.0589±0.0053 h(68.81)	0.0081±0.0006 g(82.65)
	1	0.0677±0.0031 g(79.09)	0.0083±0.0007 g(84.69)
	2	0.0845±0.0045 ef(98.72)	0.0113±0.0008 bcd(115.31)
	4	0.0693±0.0050 g(80.96)	0.0102±0.0005 def(104.08)
F		33.781	19.259
p		0.000^*^	0.000^*^

Abbreviations: SAR, simulated acid rain; Si, silicon; FW, fresh weight; DW, dry weight.

The values are the means ± standard deviation, n = 3. The value in brackets is relative percentage (the ratio of absolute value to the control).

Significant differences (*p*<0.05) are indicated by different letters in the same column.

^*^ Significant at the 0.05 level.

The FW and DW of the roots of rice seedlings treated with SAR at pH 4.0 were increased by 6.97% and 7.38%, respectively, compared with the control, but the corresponding values of the roots treated with SAR at pH 3.0 were unchanged compared with the control (Table 3). The deleterious effects of the SAR (pH 2.0) treatment on the root FW and DW were markedly stronger than those of the SAR (pH 3.0) treatment, as indicated by 31.19% and 17.35% decreases in these values, respectively, compared with the control (Table 3).

The FW and DW of the roots subjected to the combined treatments with Si (1 or 2 mM) and SAR (pH 4.0 or 3.0) were clearly increased compared with those of the control roots and the roots subjected to the corresponding single treatments with Si or SAR. The FW and DW of the roots treated with 1 mM Si and SAR (pH 2.0) were markedly decreased compared with those of the control and 1 mM Si-treated roots. In contrast, the FW and DW of the roots treated with 2 mM (4 mM) Si and SAR at pH 2.0 were markedly increased compared with those of the roots treated with SAR at pH 2.0 alone but were unchanged compared with the control and 2 mM (4 mM) Si-treated roots. In the presence of a high concentration of Si (4 mM), the roots of seedlings treated with SAR at pH 3.0 or 4.0 showed better growth than those treated with SAR at pH 2.0. The two-way ANOVA results showed an obvious interaction between Si and SAR that affected the root FW and DW of rice seedlings.

Table 4 shows the correlation coefficients between root phenotypic traits and FW or DW of the roots of rice seedlings treated with Si and SAR. The results indicated that the TRL, RSA, RV and RTN were positively correlated with the root FW and DW (*p* < 0.05), but no correlation between the AD and root FW and DW was found.

**Table 4:** Relationship of four root phenotypic traits and Si concentration with the FW or DW of the roots of rice seedlings treated with Si and SAR.

FW		DW	
Linear regression equation	Correlation coefficient (*R*)	Linear regression equation	Correlation coefficient (*R*)
Y_1_ = 209.065X_1_-10.730	0.857^**^	Y_1_ = 1786.497X_2_-11.263	0.628^**^
Y_2_ = 149.933X_1_-2.154	0.926^**^	Y_2_ = 1350.104X_2_-3.299	0.763^**^
Y_3_ = 4.304X_1_-0.079	0.935^**^	Y_3_ = 38.526X_2_-0.110	0.761^**^
Y4 = 2187.111X_1_+10.271	0.755^**^	Y4 = 21374.533X_2_-25.011	0.743^**^
Y_5_ = 1789.5421X_1_-58.469	0.768^**^	Y_5_ = 18657.209X_2_-100.260	0.870^**^

Abbreviations: SAR, simulated acid rain; Si, silicon; FW, fresh weight; DW, dry weight; TRL, total root length; RSA, root surface area; RV, root volume; RTN, root tip number.

X_1_ and X_2_ represent the root FW and DW, respectively.

Y_1_, Y_2_, Y_3_, Y_4_ and Y_5_ represent the RTL, RSA, RV, RTN and Si concentration, respectively.

^**^ Significant at the 0.01 level.

### Effects of Si on the concentrations of mineral elements in rice seedling roots under SAR

The concentrations of macroelements (K, Ca, and Mg) and microelements (Cu, Zn, and Fe) in the roots of rice seedlings treated with Si and SAR are presented in Table 5. The concentrations of Mg and Fe in the roots treated with 1 mM Si were decreased compared with the control, and a greater reduction was observed in roots treated with 2 or 4 mM Si (Table 5). The root Ca concentration was decreased after treatment with low (1 mM) and moderate (2 mM) concentrations of Si but increased after treatment with a high concentration of Si (4 mM). The opposite effect was observed for the Zn concentration (Table 5). Compared with the control, the Cu concentrations in the roots was decreased by treatment with 2 mM Si alone, and the 1 and 4 mM Si single treatments exerted a stronger effect (Table 5). The K concentration in the roots of rice seedlings was increased after treatment with the moderate (2 mM) concentration of Si but decreased after treatment with a low or high (1 or 4 mM) concentration (Table 5).

**Table 5:** Effects of Si and SAR on mineral element concentrations in the roots of rice seedlings.

SAR (pH)	Si (mM)	Macroelements			Microelements			Si μg·g^-1^(DW)
		K mg·g^-1^(DW)	Ca mg·g^-1^(DW)	Mg mg·g^-1^(DW)	Cu μg·g^-1^(DW)	Zn μg·g^-1^ (DW)	Fe μg·g^-1^(DW)	
6.5	0	5.835±0.074 i	2.973±0.018 c	2.696±0.011 b	38.900±0.400 g	28.067±0.289 j	542.900±4.004 f	81.167±0.115 h
	1	4.100±0.061 l	1.486±0.004 j	2.437±0.006 e	22.533±0.289 m	32.000±0.001 h	526.233±12.057 g	92.833±1.801 g
	2	6.017±0.050 hi	1.950±0.011 ghi	2.352±0.007 f	35.033±0.404 i	30.633±0.231 I	454.033±7.565 h	107.167±4.050 f
	4	1.918±0.046 m	3.169±0.044 b	2.131±0.017 h	26.267±0.231 l	25.433±0.231 l	271.177±2.203 l	90.700±3.923 g
4.0	0	4.343±0.088 k	4.223±0.043 a	2.147±0.013 h	31.133±0.611 j	46.167±0.289 c	596.633±1.904 e	115.767±2.761 e
	1	8.873±0.337 e	4.252±0.045 a	2.582±0.016 c	36.333±0.666 h	23.667±0.231 n	642.477±8.851 d	187.967±1.986 b
	2	9.829±0.101 d	4.261±0.005 a	2.490±0.045 d	25.767±0.462 l	18.500±0.001 p	542.400±1.100 f	195.600±4.139 a
	4	1.647±0.015 n	3.189±0.003 b	2.282±0.005 g	29.200±0.001 k	19.833±0.231 o	285.777±1.124 k	121.433±4.051 e
3.0	0	8.093±0.077 f	1.978±0.017 gh	2.070±0.005 i	26.033±0.577 l	27.300±0.001 k	766.733±4.302 a	65.333±0.289 j
	1	14.859±0.132 b	1.505±0.018 j	2.300±0.015 g	69.500±0.656 d	49.767±0.231 a	717.277±6.568 b	104.733±0.289 f
	2	16.775±0.108 a	1.471±0.006 j	2.911±0.041 a	38.933±0.513 g	35.100±0.721 e	653.700±5.730 c	150.533±6.902 c
	4	10.936±0.070 c	1.924±0.028 i	2.440±0.019 e	66.700±0.557 e	32.933±0.231 g	534.333±1.365 fg	125.933±5.085 d
2.0	0	4.191±0.037 kl	1.985±0.010 g	1.361±0.015 l	87.200±0.346 a	39.100±0.001 d	412.500±14.728 j	48.200±1.905 l
	1	5.163±0.061 j	2.601±0.052 d	2.018±0.003 j	72.867±0.462 c	33.833±0.231 f	434.103±2.771 i	57.667±1.518 k
	2	6.136±0.081 h	2.403±0.006 f	2.309±0.015 g	40.900±0.557 f	46.733±0.231 b	457.033±5.950 h	78.800±5.615 hi
	4	7.634±0.116 g	2.534±0.012 e	1.970±0.009 k	85.100±0.400 b	24.667±0.231 m	404.733±2.312 j	74.333±1.266 i
F		25.232	26.209	11.234	15.715	4.157	42.397	38.997
p		0.000^*^	0.000^*^	0.000^*^	0.000^*^	0.000^*^	0.000^*^	0.000^*^

The values are the means ± standard deviation, n = 3.

Significant differences (*p*<0.05) are indicated by different letters in the same column.

^*^ Significant at the 0.05 level.

The analysis of the single SAR treatments revealed that the K concentration in the roots of rice seedlings was increased by SAR at pH 3.0 but decreased by SAR at pH 4.0 or 2.0. In addition, the Ca concentration was increased by SAR at pH 4.0 but decreased by SAR at pH 3.0 or 2.0, and the Mg concentration was decreased compared with the control. The microelement analysis demonstrated that compared with the control, the Cu and Zn concentrations were decreased by SAR at pH 3.0 but increased by SAR at pH 4.0 or 2.0, and the Fe concentration was decreased by SAR at pH 2.0 but increased by SAR at pH 4.0 or 3.0 (Table 5).

The analysis of the combined treatments with SAR and Si showed that the root K concentration of rice seedlings treated with 1 or 2 mM Si and SAR at pH 3.0 was higher than those of the control seedlings, the seedlings subjected to the corresponding single Si treatment, the seedlings subjected to SAR alone and the seedlings subjected to the other combined treatments. Similar results were found for the Mg concentration, whereas the opposite effect was found for the Ca concentration. The K concentration in the roots of rice seedlings treated with 4 mM Si and SAR at pH 3.0 was higher than those of the corresponding single 4 mM Si-treated roots and the corresponding single SAR (pH 3.0)-treated roots, and similar findings were obtained for the Mg concentration. Conversely, the opposite effect was observed for the Ca concentration. The Zn concentration of the roots treated with Si (1, 2, or 4 mM) and SAR at pH 4.0 was decreased compared with the controls roots, the roots subjected to the single Si treatments, the single SAR (pH 3.0 or 2.0) treatments and the combined treatments with Si and SAR at pH 3.0 or 2.0. A similar effect was observed for the Cu concentration, even though the Cu concentrations with Si and SAR at pH 4.0 were lower. The analysis of the Fe concentration showed that at the same SAR level, the patterns of the changes obtained with the combined treatments were similar to those obtained with the single Si treatments. At different Si levels, the changing trends obtained for the Fe concentration with the combined treatments were similar to those obtained with the SAR single treatments. A two-way ANOVA revealed an obvious interaction between Si and SAR that affected the concentrations of macroelements and microelements in the roots of rice seedlings under these treatments.

### Effects of Si on the Si concentration in rice seedling roots under SAR

The root Si concentration of rice seedlings treated with Si were significantly increased compared with the control, and the highest value was obtained with 2 mM Si. The Si concentration in the roots of rice seedlings treated with 4 mM Si was decreased compared with that of the seedlings treated with 2 mM Si (Table 5).

Under SAR at pH 4.0, the root Si concentration was significantly increased. In contrast, the root Si concentration of seedlings treated with SAR at pH 3.0 or 2.0 was significantly decreased compared with the control (Table 5).

The Si concentrations of the roots subjected to the combined treatments with Si and SAR at pH 4.0 or 3.0 were increased compared with the control roots and the roots subjected to the corresponding single treatments with Si and SAR (pH 4.0 or 3.0). Although the Si concentration in the roots of rice seedlings treated with Si and SAR at pH 2.0 was increased compared with that of the roots treated with SAR at pH 2.0, this concentration was decreased compared with the control and Si-treated roots. Greater increases were observed with the combined treatment with 2 mM Si and SAR at increasing pH values. Two-way ANOVA results indicated an interaction between Si and SAR that affected the Si concentration in the roots of rice seedlings treated with Si and SAR. Table 4 shows the correlation coefficients between the Si content and FW or DW of the roots of rice seedlings treated with Si and SAR. The results indicated that the Si concentration was positively correlated with the root FW and DW (*p* < 0.05).

### Effects of Si on H_2_O_2_ content in rice seedling roots under SAR

The analysis of the single treatments with Si revealed that compared with the control, the H_2_O_2_ contents in the roots of rice seedlings treated with 1 or 2 mM Si were decreased, but the H_2_O_2_ contents in the roots of rice seedlings treated with 4 mM Si were increased (Table 6). Increases in the concentration of Si resulted in gradual increases in the H_2_O_2_ content.

**Table 6:** Effects of Si and SAR on CAT, POD, SOD, and APX activities and H_2_O_2_ content in rice roots.

SAR (pH)	Si (mM)	H_2_O_2_ μmol·g-1(FW)	CAT U·g^-1^(FW)·min^-1^	POD U·g^-1^(FW)·min^-1^	SOD U·g^-1^(FW)	APX U·g^-1^(FW)·min^-1^
6.5	0	0.257±0.003 j	10.717±0.282 e	5.247±0.143 d	733.122±30.821 hi	26.556±1.020 ef
	1	0.216±0.002 m	2.525±0.070 i	4.674±0.147 ef	729.872±49.983 hi	25.509±1.069 f
	2	0.249±0.003 k	2.327±0.028 i	3.647±0.096 i	795.163±25.556 gh	27.744±2.239 def
	4	0.306±0.005 h	2.639±0.066 i	4.738±0.060 e	927.328±26.781 de	24.475±2.569 fg
4.0	0	0.238±0.003 l	16.151±0.271 b	3.757±0.095 i	673.442±26.393 ijk	28.339±2.342 def
	1	0.189±0.002 n	7.445±0.169 g	4.300±0.048 h	651.427±13.041 jk	43.282±2.345 b
	2	0.193±0.002 n	9.815±0.249 f	4.496±0.150 g	642.085±22.597 k	51.182±2.501 a
	4	0.284±0.002 i	7.533±0.224 g	1.583±0.064 l	708.695±29.520 ij	29.902±2.327 de
3.0	0	0.387±0.005 e	5.516±0.147 h	5.674±0.047 c	834.339±35.554 fg	19.797±2.501 h
	1	0.351±0.003 f	10.654±0.653 e	4.193±0.060 h	1318.142±35.187 b	31.305±2.216 d
	2	0.325±0.003 g	19.423±0.517 a	4.503±0.071 fg	1472.469±60.287 a	36.715±1.950 c
	4	0.385±0.005 e	13.478±0.239 c	2.223±0.111 i	988.815±42.872 d	28.147±1.497 def
2.0	0	0.512±0.004 a	1.986±0.061 i	2.001±0.066 k	874.758±26.359 ef	12.127±0.661 i
	1	0.431±0.006 c	10.257±0.223 ef	6.072±0.058 b	1066.686±41.675 c	18.778±1.432 h
	2	0.394±0.005 d	12.404±0.356 d	8.636±0.213 a	1122.326±54.544 c	20.576±1.647 gh
	4	0.465±0.004 b	9.914±0.168 f	4.176±0.056 h	953.960±52.680 d	14.243±1.693 i
F		178.961	4.336	4.176	20.131	38.219
p		0.000^*^	0.002^*^	0.002^*^	0.000^*^	0.000^*^

Abbreviations: SAR, simulated acid rain; Si, silicon; CAT, catalase; POD, peroxidase; SOD, superoxide dismutase; APX, ascorbate peroxidase; H_2_O_2_, hydrogen peroxide.

The values are the means ± standard deviation, n = 3.

Significant differences (*p*<0.05) are indicated by different letters in the same column.

^*^ Significant at the 0.05 level.

The investigation of the single SAR treatments demonstrated that compared with the control, the H_2_O_2_ contents in the roots of rice seedlings treated with SAR at pH 4.0 were decreased, but H_2_O_2_ contents in the roots of rice seedlings treated with SAR at pH 3.0 or 2.0 were increased (Table 6). Decreases in the pH value of acid rain resulted in clear increases in the H_2_O_2_ contents.

The analysis of the combined treatments with Si and SAR revealed that the application of 1 or 2 mM Si decreased the H_2_O_2_ contents in the roots of rice seedlings treated with SAR at pH 4.0 compared with the roots of rice seedlings subjected to the corresponding single treatments with Si and SAR and the control roots. However, the rice seedlings treated with SAR at pH 4.0 and 4 mM Si presented higher H_2_O_2_ contents compared with the SAR (pH 4.0)-treated and control seedlings, but these contents were lower than those of the 4 mM Si-treated seedlings. The supply of Si significantly decreased the H_2_O_2_ contents in the roots of rice seedlings treated with SAR at pH 3.0 or 2.0 compared with the corresponding single SAR treatment. Increases in the concentration of Si first decreased and then increased the H_2_O_2_ contents.

### Effects of Si on the activities of antioxidative enzymes in rice seedling roots under SAR

Table 6 shows the changes in antioxidative enzyme activities in the roots of rice seedlings treated with Si and SAR, with the significant differences noted. Superoxide dismutase (SOD) activity in the roots was not significantly changed by the supply of 1 or 2 mM Si, even though significant increases were observed between the high concentration of Si (4 mM) and the control. Si significantly decreased POD activity compared with the control. In particular, the supply of 2 mM Si decreased POD activity in rice seedlings compared with the control and the corresponding 1 or 4 mM Si single treatment. A similar result was found for CAT activity. In contrast, the supply of Si to rice seedlings had no discernible effect on root APX activity.

The analysis of single SAR treatments revealed that exposure to SAR at pH 4.0 decreased SOD and POD activities but increased CAT activity compared with the control, whereas APX activity remained unchanged. SAR at pH 3.0 increased SOD and POD activities in roots compared with the control and SAR (pH 4.0) alone. The opposite effect was observed for CAT and APX activities. POD, CAT, and APX activities in roots treated with SAR at pH 2.0 were lower than those of the control roots and the SAR (pH 4.0 or 3.0)-treated roots.

The combined treatments with Si and SAR revealed similar trends for the changes in SOD, POD, CAT and APX activities in roots. The supply of Si significantly increased SOD, APX, CAT and POD activities in the roots of rice seedlings treated with moderate or severe SAR (pH 3.0 or 2.0) compared with the corresponding single SAR treatment (pH 3.0 or 2.0). POD activity in the roots of rice seedlings treated with Si and severe SAR (pH 2.0) was significantly increased compared with that observed in the seedlings subjected to SAR at pH 2.0, and APX activity in the roots of seedlings treated with SAR at pH 4.0 or 2.0 was higher than that obtained with all of the other treatments.

## Discussion

The phenotype and biomass of plant roots display marked changes in response to environmental stress [11,50,60–63], and our experimental results showed the effects of Si and SAR on the roots of rice seedlings. First, the roots of rice seedlings treated with a low or moderate concentration of Si (1 or 2 mM) showed enhanced growth. In particular, the root phenotype and biomass of rice seedlings simultaneously treated with a low or moderate concentration of Si and light or moderate SAR (pH 4.0 or 3.0) were improved compared with those of rice seedlings exposed to the corresponding Si or SAR single treatments. Second, treatment with a high concentration of Si (4 mM) or severe SAR (pH 2.0) had negative effects on root phenotype and biomass (Fig 1 and Table 2); in addition, the phenotype and biomass of the roots of rice seedlings treated with a high concentration of Si (4 mM) and SAR were improved compared with the roots treated with a high concentration of Si (4 mM) and the roots treated with severe SAR (pH 2.0). These findings indicate that the inhibitory effects of a high concentration of Si (4 mM) or severe SAR (pH 2.0) on the root phenotype and biomass of rice seedlings were alleviated by the other treatment. Third, the influence of the supply of Si on the root phenotype and biomass depended on the Si concentration and the SAR intensity (Fig 1 and Table 2), and two-way ANOVA results indicated an obvious interaction between Si and SAR that affected the root phenotypic traits and biomass of rice seedlings (Tables 2 and 3). However, whether this interaction is additive or synergistic remains unclear, and this question should be addressed in future work.

Si plays a vital role in plants. One of the main functions of Si is to increase plant growth and yield, particularly under conditions of stress [22,64,65]. Studies have shown that different concentrations of Si caused different effects [63–65]. The correlation analysis performed in this study shows that the Si concentration in roots of rice seedings treated with Si and SAR is positively correlated with the root FW and DW (Table 4). The incorporation of Si (1, 2 or 4 mM) into SAR boosted root growth and increased the Si concentration in the roots of rice seedlings compared with the corresponding SAR single treatment. However, the addition of a high concentration of Si (4 mM) restricted root growth compared with the low or moderate concentration of Si (Tables 3 and 5) because Si deposition in rice roots decreases the ability of roots to absorb Si [66]. The results of the present study also support this conclusion (Tables 3 and 5). Perry reported that silica condensation in nature is affected by many factors, including the silica concentration, pH, and temperature, as well as the presence of other polymers, small molecules, and different ions [67]. SAR could relieve the toxicity detected in the roots of rice seedlings exposed to a high concentration of Si (4 mM; Table 3), which could be due to the fact that a low pH inhibits Si deposition [67]. However, this mechanism needs to be further studied.

Root growth directly influences the root phenotype. This study demonstrated that the phenotype of roots treated with Si and SAR is positively correlated with the root biomass (Table 4). Root growth is influenced by nutrient absorption [50,68]. K, Ca, Mg, Cu, Zn and Fe are considered essential elements for plant growth and development. K is a major contributor to the metabolic function and organic structure of plants due to its effect on protein synthesis, enzyme activation and photosynthesis [69]. Ca is a major plant nutrient that affects the maintenance of cell wall structure and membrane function. As a second messenger for the transduction of stress signals, Ca regulates the physiological and biochemical responses in plant [70]. The negative effects of SAR on seed germination, seedling growth and photosynthesis of four sensitive species could be ameliorated by the addition of Ca. Previous studies have demonstrated that different species often show various capacities of tolerance to acid rain due to their different Ca requirements [71]. As an enzyme cofactor and an important component of chlorophyll, Mg plays a central role in plant chlorophyll biosynthesis and carbon fixation [72]. Microelements (Cu, Zn and Fe) play important roles in the structure, function and metabolic activity of plants [73]. Previous studies have shown that nutrient elements can affect plant yield and growth to varying extents [74,75]. Therefore, the maintenance of adequate levels of nutrient elements is essential for plant growth and survival under environment stresses. Some studies have shown that acid rain disturbs the absorption and utilization of nutrient elements and thereby affects plant growth [11,50]. Our experiments showed similar results (Table 5), but the disturbances caused by SAR were affected by the addition of Si. In particular, treatment with severe SAR (pH 2.0) decreased the rice root biomass (FW and DW), significantly decreased the K, Ca, Mg, Fe and Si concentrations and clearly enhanced the Cu and Zn concentrationts. The incorporation of Si (1, 2 or 4 mM) into severe SAR (pH 2.0) significantly increased the rice root biomass (FW and DW) and relieved any sharp increases or decreases (Table 5). Our experimental results indicated that Si in the roots of rice seedlings or Si treatment disturbed the absorption and utilization of these nutrients (K, Ca, Mg, Cu, Zn and Fe) by the roots and maintained the relative balance of the mineral element contents in roots. Similar results have been reported previously [22,30], and changes in the concentrations of minerals might constitute a mechanism for inhibiting or promoting root growth [61,74,76].

Under environmental stress, reactive oxygen species (ROS), including H_2_O_2_, are accumulated in plants [11,77]. In the present study, a similar response was observed in the roots of rice seedlings treated with SAR. Moreover, the incorporation of Si into SAR resulted in decreased H_2_O_2_ content (Table 6). Previous studies indicated that the addition of Si decreased the H_2_O_2_ concentration in plants under a stress environment [78,79], indicating that Si alleviates oxidative stress induced by acid rain.

ROS caused by environmental stress can be removed by antioxidant enzymes, including SOD, POD, CAT, and APX [9,11,77,80,81]. SOD is crucial for removing ROS [82]. In the present study, SOD activity was found to be significantly enhanced in the roots of rice seedlings exposed to moderate or severe SAR (pH 3.0 or 2.0; Table 6), implying an important role for SOD in remitting oxidative stress induced by SAR. Under moderate or severe SAR (pH 3.0 or 2.0), the addition of Si enhanced SOD activity compared with the corresponding SAR single treatment (Table 6), indicating increased scavenging. Similar results have showed that Si supplementation alleviates salt stress by increasing antioxidant enzyme activity in plants [18,83]. CAT is one of the key enzymes involved in the removal of toxic peroxides through the decomposition of H_2_O_2_ into O_2_ and H_2_O. A decline in CAT activity under moderate or severe SAR (pH 3.0 or 2.0) was observed in the present investigation (Table 6), and a similar decrease was previously reported [43]. However, Si significantly reversed the decrease in CAT activity observed in the roots of seedlings treated with SAR (Table 6), indicating that Si increases the amount of antioxidants or scavengers and thereby reduces radicals, as was previously shown [11,30,50,84]. POD also plays an important role in scavenging H_2_O_2_ in plants [77,81]. In the present study, POD activity was found to be increased in the roots of rice seedlings treated with moderate SAR (pH 3.0) but decreased in the roots treated with severe SAR (pH 2.0), and the addition of Si increased POD activity under severe SAR (pH 2.0) stress. APX plays an important role in the ascorbate-glutathione cycle and can remove H_2_O_2_ [85]. The addition of Si significantly increased APX activity under SAR stress compared with the single SAR treatment (Table 6), indicating that Si supplementation could enhance tolerance to oxidative stress under acid rain toxicity by increasing the efficiency of the ascorbate-glutathione cycle. These data indicate that Si treatment increases SOD, POD, CAT and APX activities in the roots of rice seedlings treated with moderate or severe SAR (pH 3.0 or 2.0) and decreases the H_2_O_2_ content (Table 6), suggesting that Si protects plants against oxidative stress caused by SAR. Therefore, the induced increases in antioxidant enzyme activities can be considered an important mechanism in the cellular defence strategy against oxidative stress [9,11,79–81].

Previous studies have suggested that Si, as a beneficial nutrient, might be involved in metabolic, physiological and/or structural activities in higher plants exposed to abiotic and biotic stresses [28,29], and the results of the present study support this conclusion. In summary, on the one hand, under acid rain stress, the addition of Si at a safe concentration (1 and 2 mM) improves the root phenotype and growth, increases the activities of SOD, APX, CAT and POD, influences the absorption and utilization of mineral nutrients, and relieves acid rain toxicity in the roots of rice seedlings. On the other hand, acid rain also ameliorates the toxicity of a high concentration of Si (4 mM) in roots by increasing antioxidative activities and disturbing the absorption and utilization of mineral nutrients.

## Supporting information

S1 File Enzymes activities and H_2_O_2_ content. (SAV)

S2 File Mineral element concentrations. (SAV)

S3 File Root phenotypic traits, FW and DW. (SAV)
